# Survival Rates and Factors Affecting the Outcome Following Immediate and Delayed Implant Placement: A Retrospective Study

**DOI:** 10.3390/jcm11154598

**Published:** 2022-08-07

**Authors:** Georgios S. Chatzopoulos, Larry F. Wolff

**Affiliations:** 1 Division of Periodontology, Department of Developmental and Surgical Sciences, School of Dentistry, University of Minnesota, 515 Delaware St. SE, Minneapolis, MN 55455, USA; 2 Department of Preventive Dentistry, Periodontology and Implant Biology, School of Dentistry, Aristotle University of Thessaloniki, 54124 Thessaloniki, Greece

**Keywords:** dental implants, fresh socket, healed socket, immediate implant, immediate insertion, implant failure rate, retrospective

## Abstract

Background: Immediate implant placement into extraction sockets has become a widely acceptable treatment option to decrease treatment time and enhance esthetics. The objectives of this study were to assess and compare the survival rates of immediate and delayed implant treatment as well as to investigate the effect of patient- and site-related variables on the treatment outcome in a large-scale population-based study. Methods: Dental records of patients who received implant therapy were retrieved from the electronic records of the University of Minnesota School of Dentistry. Demographic characteristics, dental insurance status, socioeconomic status as well as medical history and tobacco use were recorded. The treatment outcome was included as a binary variable (survival/failure). Time to failure (date of procedure to date of visit with failure) was compared between immediate and delayed implant treatment in Cox regression models. Kaplan–Meier plots for the survival of both treatment modalities were created. Patient-sites without failure were censored at the last follow-up visit. Results: A total of 4519 records of implants were included. The sample mean age was 60.27 years and included 50.7% males and 12.9% tobacco users. High socioeconomic status was characterized for 82.3% of the included population and 63.0% of them were self-payers. Immediate implants were significantly more frequently placed in the maxillary arch (*p* < 0.001) than in the mandible. Tobacco users received more often a delayed rather than an immediate implant placement (*p* = 0.001). The survival rate analysis revealed there were no significant differences between immediate and delayed implant placements (*p* = 0.48). The mean follow-up time was 32.27 months during which 1.5% immediate and 1.1% delayed implants were removed. The estimated mean survival time for immediate implants was 68.90 months, while delayed implants placed in healed sockets showed a mean survival time of 75.11 months. A statistically significant association was found between gender (*p* = 0.03) and osteoporosis (*p* = 0.001) with treatment outcome. Conclusions: The placement of immediate implants achieved similarly high survival rates when compared to delayed implants placed in healed sites. Males and osteoporotic individuals showed significantly higher implant failure than females and non-osteoporotic patients. This study demonstrated that both immediate and delayed implant placements are sound options with predictable treatment outcome.

## 1. Introduction

In recent decades, dental implants have become a reliable treatment option to replace missing teeth and achieve esthetics and function with good long-term prognosis [1,2]. Tooth extraction initiates a cascade of events that results in alveolar ridge reduction in the width and height [3]. This reduction is reported to be approximately 5–7 mm of the horizontal and 1 mm of the vertical bone dimension with most changes occurring within the first three months following extraction [4]. The extension of bone resorption may challenge restorative-driven implant placement or additional surgical procedures may be required prior to an ideal implant placement [5]. This may lead to increased treatment cost, morbidity, complications and treatment time [5]. Alveolar ridge preservation techniques may be performed to prevent or minimize bone resorption. Autologous bone, allografts, xenografts or newer bone regeneration techniques including equine bone substitute blocks can be used to achieve a biological and clinical outcome [6]. 

Different implant placement protocols have been introduced as a result of the technological advances in implant surface that allow quicker osseointegration and earlier bone healing than in the past [7,8]. These protocols include immediate (when a dental implant is placed immediately after a tooth extraction), early (when an implant is installed within 1–2 months following an extraction), delayed (when the placement of an implant is performed 3–4 months after an extraction), or late/conventional (when the insertion of an implant is completed at least 4 months after extraction in fully healed extraction socket) implant placements [9]. 

Following the introduction of immediate implant placement protocols, both patients and clinicians have demonstrated increased interest in this technique. Immediate implant placement is a method that decreases the number of surgeries and therefore the total treatment time [10], minimizes bone resorption following a tooth extraction and thus maintains the periodontal architecture leading to better esthetic treatment outcomes [11,12,13], and achieves optimal implant orientation and positioning [13]. In addition, this treatment approach results in higher patient satisfaction than the conventional/delayed placement protocol [14]. On the other hand, immediate implants have also been associated with increased surgical complications, poor esthetic outcomes due to gingival recessions and conflicting findings regarding their failure rates [15,16,17,18]. A dental implant is considered failed when it demonstrates clear signs or symptoms that require its removal [19]. This can either occur prior to loading and masticatory function (early implant failure) or after loading (late implant failure) [20]. Early failure is associated with poor osseointegration and the inability to achieve optimum bone to implant contact, while late failure is primarily a result of biological complications that is characterized by the inability to maintain osseointegration [19,21]. A number of risk factors of implant failure have been reported in the literature including patient-, site-, and implant-related factors [19,22].

One of the key factors for the success of implant therapy is appropriate patient selection. Therefore, it is crucial for the dental practitioner to recognize the risks of implant failure, identify patients and sites that are suitable for dental implants and a treatment plan accordingly to ensure the long-term clinical success of an implant placement. In addition, immediate implant placement into extraction sockets has become a widely acceptable treatment option to decrease the treatment time and enhance esthetics. Thus, large-scale studies should be conducted in order to investigate whether immediate implant placement is a viable treatment option when compared to delayed treatment and identify the patient and site characteristics that may influence the treatment outcome. 

The objectives of the present investigation were to assess and compare the survival rates of immediate and delayed implant treatment as well as to investigate the effect of patient- and site-related variables on the treatment outcome in a large-scale, retrospective, and population-based study.

## 2. Materials and Methods

This study was approved by the Institutional Review Board of the University of Minnesota for medical record chart review (#1606M88402). 

### 2.1. Patient Selection

The present retrospective study incorporated all consecutive patients who underwent dental implant placement and restoration at the University of Minnesota School of Dentistry clinics between 2010 and 2016. All data were retrieved from the electronic dental records of patients who fulfilled the following inclusion criteria:

- Were at least 18 years of age at the time of the treatment;
- Had complete demographic and medical history records;
- Received implant treatment in the university dental clinics provided by residents or faculty;
- Data related to implant therapy were available.

Datasheets were created using the electronic dental records of the patients including the patient’s ID number, age at the time of the implant placement, gender (male/female), ZIP code of their residence, dental insurance status (presence/absence), medical history, tobacco use, tooth/implant site and implant placement protocol (immediate or delayed). The examined systemic medical conditions were self-reported hypertension, history of heart attack, hypercholesterolemia, asthma, diabetes, thyroid disorder, kidney disease, arthritis, osteoporosis, anxiety, cancer, history of cancer therapy, and history of artificial joint replacement. With respect to the tooth/implant site characteristics, the arch (maxilla/mandible) and the region (anterior/posterior) were assessed. The socio-economic status of the patient was determined based on the ZIP code of their residence and the 2010–2014 American Community Survey 5-year estimates of the U.S Census Bureau [23]. Patients were considered to be of a lower socio-economic status when the mean annual household income of their ZIP code was below the mean value, while a mean annual household income above the mean value of the included population categorized them into a higher socio-economic status. 

### 2.2. Implant Survival and Failure

Implant failure was defined as the removal of a dental implant for any reason including the loss of osseointegration, mobility, persistent pain, fracture, and extensive bone loss as one of the most recent follow-up appointments. Implant survival was defined as the implant maintained in place and supporting the restoration at the most recent recall appointment and no indication for implant explantation was recorded. The implant failure and therefore implant removal was identified based on the ADA code: D6100 (implant removal-failure). The treatment outcome was included as a binary variable (survival/failure).

### 2.3. Statistical Analysis

Descriptive statistics including frequencies, means and standard deviations were calculated for patients’ and implant sites’ characteristics. Chi-square test and *t*-test were conducted to statistically assess the differences between the immediate and delayed implant placement treatment groups. The Kaplan–Meier plots for the survival of both treatment modalities were created. Time to failure (date of procedure to date of visit with failure) was compared between immediate and delayed implant treatment in Cox regression models. Patient-sites without a failure were censored at the last follow-up visit. An adjusted model was utilized to investigate the influence of the independent parameters on the survival of immediate and delayed implant treatments. Hazard ratios (HR) and their 95% confidence intervals (CIs) were reported for each model. All tests of significance were evaluated at the 0.05 error level with a statistical software program (SPSS v.24.0, IBM, Armonk, NY, USA).

## 3. Results

A total of 4645 dental records of implants were screened for eligibility in the study. Records of dental implants were excluded from the analysis due to incomplete data and duplicates (n = 126). Therefore, 4519 records of dental implants placed at the University Of Minnesota School Of Dentistry between 2010 and 2016 were included in the present investigation. The final sample consisted of 265 (5.9%) immediate implants and 4254 (94.1%) delayed implants. 

The demographic, site and patient characteristics of the immediate and delayed implant treatment groups as well as of the total population are shown in Table 1. The sample mean age was 60.27 years and included 50.7% males and 12.9% tobacco users. High socioeconomic status was characterized for 82.3% of the included population and 63.0% of them were self-payers (absence of dental insurance). Tobacco users received more often a delayed rather than an immediate implant placement (*p* = 0.001). Immediate implants were significantly more frequently placed in the maxillary arch than in the mandible (*p* < 0.001), while delayed implants were significantly more often placed in the posterior region than in the anterior (*p* < 0.001). No significant differences were found in regard to the treatment outcome between immediate and delayed implant placements (*p* = 0.54). With regard to the implant failure, the mean follow-up time was 32.27 months during which 1.5% immediate (n = 4) and 1.1% delayed implants (n = 47) were removed. Patients with depression were significantly more likely to receive an implant following the delayed approach (*p* < 0.001). 

**Figure 1:**
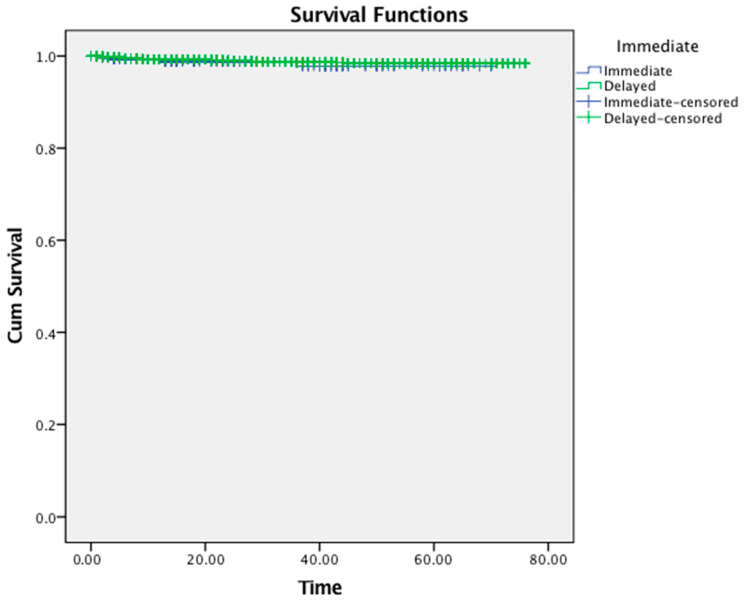
Kaplan–Meier curve showing the cumulative survival rates of immediate and delayed implant treatment to time (in months).

The cumulative survival rates of immediate and delayed implants with respect to time (in months) is shown in Figure 1. The estimated mean survival time for immediate implants was 68.90 (95% confidence interval: 67.82–69.98) months with a range of 1–70 months, while delayed implants placed in healed sockets showed a mean survival time of 75.11 months (95% confidence interval: 74.85–75.37) with a range of 1–76 months. The overall mean survival time was 75.09 (95% confidence interval: 74.84–75.34) months. The vast majority of the failed implants in the immediate placement group were removed within the first 13 months of their placement (n = 3), while the fourth one failed at 37 months. Similarly, more than half of the implant failures of the delayed group occurred within the same timeframe. Twenty-nine delayed implants failed within the first 13 months of their insertion.

The overall survival rate up to 70 months for implants placed immediately following tooth extraction was 98.5%, while implants inserted in completely healed extraction sockets showed a survival rate of 98.9% up to 76 months. No significant differences were found between the two implant treatment protocols (*p* = 0.48). The multivariable Cox regression model is summarized in Table 2. A statistically significant association was found between gender (*p* = 0.03) and osteoporosis (*p* = 0.001) with treatment outcome. Male patients were at 2.06 (hazard ratio: 2.06, 95% CI: 1.08–3.92) significantly increased risk of experiencing implant failure than female individuals (*p* = 0.03). In addition, osteoporotic patients exhibited a 4.56 (Hazzard ratio: 4.56, 95% CI: 1.80–11.54) significantly higher risk of having an implant failure than non-osteoporotic implant-treated patients (*p* = 0.001). 

## 4. Discussion

This study was undertaken to investigate the survival rates of implants placed immediately and 3–4 months (delayed approach) following a tooth extraction as well as to identify the potential risk indicators that are associated with implant failures. For this purpose, we conducted a large-scale, retrospective, population-based study utilizing the electronic dental records of the University of Minnesota School of Dentistry. A total of 4519 records of dental implants were included in the analysis and our main findings were:

- The overall survival rate of implants placed immediately following tooth extraction was 98.5%, while implants inserted in completely healed extraction sockets showed a survival rate of 98.9%. Nevertheless, imbalances in the baseline characteristics such as tobacco use, arch, region and depression due to study design may have partially influenced the reported finding;
- The implant treatment protocol (immediate versus delayed) was not associated with a higher risk of implant failure (*p* = 0.48);
- Male patients (*p* = 0.03) and individuals with osteoporosis (*p* = 0.001) were more likely to experience dental implant failure than females and non-osteoporotic patients, respectively;
- Implants failed predominantly within the first 13 months of their placement with no differences between the treatment groups.

A challenging dilemma in implant dentistry is when to place a dental implant immediately following an extraction or to opt for a delayed placement once the soft and hard tissues have healed. The literature remains controversial. Implant treatment protocols have shown both failures and complications. A randomized clinical trial that compared the clinical outcomes of single implants placed immediately after an extraction, with implants inserted at 6 weeks (immediate-delayed) or at 4 months demonstrated no significant differences with respect to failure rates, complications and patient satisfaction [24]. Immediate and immediate–delayed implants resulted in better esthetic results than delayed implants when the total esthetic score was used [24]. In contrast, a randomized clinical trial that included patients in need of a single implant in the anterior and premolar areas reported higher radiographic bone loss, deeper probing depths and more frequent inadequate pink esthetic scores in subjects that received immediate implants than those subjects who received one at 12 weeks [25]. In addition, in a prospective multicenter clinical study with a follow-up of 3 years that included 264 implants, both immediate and delayed implants demonstrated similar cumulative survival rates [26]. Similar results were also found for single-tooth replacement in the esthetic zone as well as in the maxillary molar region when implants were inserted immediately or after extraction socket healing [27,28]. The timing of implant placement did not affect the survival rates of single-tooth implants when both tapered and cylindrical implants were used and assessed 5 years after loading [29]. Our findings agree with these investigations. 

A number of systematic reviews have been conducted evaluating this topic. Mello et al. searched the literature and identified 30 studies published until November 2016 with a minimum follow-up time of 6 months [30]. They concluded that immediate implants exhibited a significantly higher failure rate than delayed implants and they reported that immediate implant placement should be used with caution [30]. In parallel with these findings, another systematic review and meta-analysis that included randomized and non-randomized studies up to May 2018 with at least 1 year of follow-up showed significantly lower implant survival for immediate placement as compared to delayed [31]. No differences were detected between the treatment groups for probing pocket depth and the pink esthetic score [31]. In contrast, when the success of single immediate implants was assessed, high success rates, patient satisfaction and esthetics were reported with limited biological and hardware complications [32]. Moreover, a meta-analysis that included 163 publications in which 17,278 and 38,738 implants placed in fresh extraction sockets and healed sites, respectively, showed an increased risk of implant failure in fresh sockets and more specifically in the maxillary implants [33].

In addition, we investigated variables that might affect the survival rate of dental implants placed immediately or delayed following a tooth extraction. Patients of a different gender exhibited significantly different survival rates, with males showing a 2.06 significantly higher risk of implant failure than females (*p* = 0.03). The influence of gender on implant survival is controversial in the literature. In agreement with our study, male patients were associated with a 1.97 (95% CI: 1.42–2.75) higher risk of early and late implant loss in a retrospective study that included 30,959 records of dental implant procedures in China [34]. Another retrospective study demonstrated that men were 1.65 times more likely to experience implant failure than females [35] and other studies support these findings [36,37]. In contrast, other publications report no significant differences between male and female patients, while the diagnosis of periodontal disease may play an important role for implant survival [22,38,39,40]. Data on the periodontal status of these patients were not available for the present investigation and therefore periodontal disease could not be assessed as a predictor of implant loss. This may be considered a limitation of our investigation. 

Various studies have examined the effect of systemic medical conditions on the survival rates of dental implants. Although osteoporosis, human immunodeficiency virus, cardiovascular disease, hypothyroidism, bleeding disorders and diabetes have been identified as conditions that may affect implant survival, the available literature is inconclusive [41,42]. The present study found a significantly higher risk of implant failure in patients with osteoporosis than non-osteoporotic individuals (*p* = 0.001). Osteoporosis was among other factors associated with an increased risk of implant failure in a recent retrospective case–control study that evaluated records of implant removal [43]. Similar findings were also reported in another study in which osteoporotic patients were more likely to experience early implant failure than those who were non-osteoporotic [44]. Systematic reviews have shown controversial findings in regard to the impact of osteoporosis in implant treatment outcome and therefore additional research is needed [45,46,47]. It is worth noting that the differences between various studies may be attributed to the inclusion of patients with a different severity/degree of systemic disease-control [48]. Furthermore, in our analysis, none of the other examined systemic conditions were associated with implant failure. This is in agreement with a long-term, hospital-based study which concluded that no systemic disease or condition increased the risk for implant failure [49].

In general, various factors have been investigated in the literature for their role in implant survival including age, gender, implant length and diameter, implant location, patient’s medical condition, smoking habits, implant location as well as bone quality [50,51,52,53]. In a large retrospective study of 30,959 implants, Lin and colleagues demonstrated that males, patients aged ≥ 41 years, and mandibular anterior location were risk factors for early implant loss, whereas males, patients aged ≥ 41 years, bone augmentation, and short implants were risk factors for late implant loss [34]. Smoking has a detrimental effect on implant survival that is mainly attributed to the lower bone formation rate and longer mineralization time as well as the abnormal angiogenesis that leads to decreased vascularization and remodeling [53,54]. A recent systematic review and meta-analysis showed that implants in smokers exhibited a 140.2% higher failure risk when compared to non-smokers as well as an increased marginal bone loss [55]. In the present investigation, tobacco use did not affect the outcome following immediate and delayed implant placement. 

The association of implant survival with bone augmentation is yet unclear. Carr and colleagues have shown that implants placed in augmented areas are 5-fold more likely to fail compared to implants placed in non-grafted areas [56]. On the other hand, studies have reported that implants in grafted sinuses exhibited a 97.5% survival rate compared to 90.3% for implants placed in native bone in the posterior maxilla) [57,58]. In addition, a retrospective study with a long-term follow-up time depicted that implants placed in areas with regenerated bone exhibited predictable clinical results and no differences were detected between the source of the graft (autologous or demineralized bovine bone) [59]. Similarly, in another retrospective study, the 5-year cumulative survival rate of implants placed with guided bone regeneration was similar to those placed in native bone [60]. These variables were not included in the present study because of the limited available information, and this is considered a weakness of this study.

A different parameter that has been associated with a possible higher implant failure in immediately placed dental implants is the presence of apical periodontitis. Early studies reported that immediate implants are contraindicated in the case of periapical and periodontal lesions due to the risk of microbial interference [61,62]. Recent evidence suggests that immediate implants in sites with periapical and periodontal pathology results in comparable clinical outcomes compared to those placed in healthy sites providing that meticulous cleaning, socket curettage/debridement, and chlorhexidine 0.12% rinse are performed prior to implant placement [63,64,65]. Due to the retrospective design of the present study, no such data were available for the included patients’ records.

The retrospective design of the present study should be considered when interpreting the findings. The variety of treatment providers and the lack of information with respect to periodontal status as well as the potential bone augmentation procedures are limitations of the present investigation which may inherently lead to flaws. However, no differences in the survival rates have been reported when implants are placed in native or bone-grafted sites [66]. Due to the retrospective design of the investigation and the use of a large number of electronic records of dental implants placed at a university clinic, it was not possible to report implant success rates and assess the implants clinically and radiographically. Implants are considered successful if there is less than 0.2 mm bone loss annually after the first year of loading, if they are clinically immobile, if there is no peri-implant radiolucency and if there is no persistent and/or irreversible pain, infection, neuropathies or paresthesia [67]. For the present investigation, no radiographs were available for the evaluation of the peri-implant bone and therefore implant success could not possibly be assessed. Implant survival was instead utilized in the present study as the primary outcome. Implant failure was defined as the removal of a dental implant for any reason including loss of osseointegration, mobility, persistent pain, fracture, and extensive bone loss as of the most recent follow-up appointment. Implant survival was defined as the implant maintained in place and supporting the restoration at the most recent recall appointment and no indication for implant explantation was recorded. 

A strength of this study was the evaluation of a large number of dental records of implants placed at a university dental clinic following the evidence-based surgical and prosthetic implant protocols which increase the validity of our findings and eliminate the selection bias. Further data must be provided that reflect the actual performance in the daily dental practice. Further prospective studies are needed to assess the survival rates of immediate, early and delayed implants and to investigate the risk factors associated with implant failure in order to be able to provide individualized implant treatment plans and achieve optimum treatment outcomes. 

## 5. Conclusions

Within the limitations of the present large retrospective study, the cumulative survival rates of 98.5% for implants placed immediately following an extraction and 98.9% for implants inserted in healed sockets reveal no significant differences. The results suggest that male and osteoporotic patients were associated with a higher risk of implant failure. Further well-designed prospective studies are needed. 

## Figures and Tables

**Table 1:** Demographic, site and patient characteristics of the immediate and delayed implant treatment groups as well as of the total population.

Characteristics	Total	Immediate Implant	Delayed Implant	*p*-Value *
	n = 4519—100%	n = 265—5.9%	n = 4254—94.1%	
Age	60.27 ± 13.11 (Range: 18–93)	59.89 ± 14.10	60.30 ± 13.05	0.62
Gender n (%)				0.31
Males	2293 (50.7)	126 (47.5)	2167 (50.9)	
Females	2226 (49.3)	139 (52.5)	2087 (49.1)	
Tobacco use n (%)				**<0.001**
Yes	581 (12.9)	16 (6.0)	565 (13.3)	
No	3938 (87.1)	249 (94.0)	3689 (86.7)	
Socioeconomic status				0.87
n (%)				
Low	800 (17.7)	48 (18.1)	752 (17.7)	
High	3719 (82.3)	217 (81.9)	3502 (82.3)	
Insurance status n (%)				0.27
Yes	2849 (63.0)	176 (66.4)	2673 (62.8)	
No	1670 (37.0)	89 (33.6)	1581 (37.2)	
Arch n (%)				**<0.001**
Maxilla	2333 (51.6)	173 (65.3)	2160 (50.8)	
Mandible	2186 (48.4)	92 (34.7)	2094 (49.2)	
Region n (%)				**<0.001**
Anterior (incisors, canines)	1285 (28.4)	132 (49.8)	1153 (27.1)	
Posterior (premolars, molars)	3234 (71.6)	133 (50.2)	3101 (72.9)	
Treatment outcome				0.54
n (%)				
Survived	4468 (98.9)	261 (98.5)	4207 (98.9)	
Failed	51 (1.1)	4 (1.5)	47 (1.1)	
Hypertension n (%)				0.36
Yes	1246 (27.6)	66 (24.9)	1180 (27.7)	
No	3273 (72.4)	199 (75.1)	3074 (72.3)	
History of heart attack n (%)				0.38
Yes	151 (3.3)	6 (2.3)	145 (3.4)	
No	4368 (96.7)	259 (97.7)	4109 (96.6)	
High cholesterol				0.56
n (%)				
Yes	1166 (25.8)	64 (24.2)	1102 (25.9)	
No	3353 (74.2)	201 (75.8)	3152 (74.1)	
Asthma				0.52
Yes	288 (6.4)	14 (5.3)	274 (6.4)	
No	4231 (93.6)	251 (94.7)	3980 (93.6)	
Diabetes n (%)				0.14
Yes	386 (8.5)	16 (6.0)	370 (8.7)	
No	4133 (91.5)	249 (94.0)	3884 (91.3)	
Thyroid disorder				0.38
n (%)				
Yes	534 (11.8)	36 (13.6)	498 (11.7)	
No	3985 (88.2)	229 (86.4)	3756 (88.3)	
Kidney disease n (%)				0.35
Yes	85 (1.9)	7 (2.6)	78 (1.8)	
No	4434 (98.1)	258 (97.4)	4176 (98.2)	
Arthritis n (%)				0.25
Yes	1001 (22.2)	51 (19.2)	950 (22.3)	
No	3518 (77.8)	214 (80.8)	3304 (77.7)	
Artificial joint n (%)				0.25
Yes	363 (8.0)	16 (6.0)	347 (8.2)	
No	4156 (92.0)	249 (94.0)	3907 (91.8)	
Osteoporosis n (%)				0.89
Yes	251 (5.6)	15 (5.7)	236 (5.5)	
No	4268 (94.4)	250 (94.3)	4018 (94.5)	
Depression n (%)				**0.001**
Yes	604 (13.4)	18 (6.8)	586 (13.8)	
No	3915 (86.6)	247 (93.2)	3668 (86.2)	
Anxiety n (%)				0.23
Yes	505 (11.2)	23 (8.7)	482 (11.3)	
No	4014 (88.8)	242 (91.3)	3772 (88.7)	
Cancer n (%)				0.92
Yes	520 (11.5)	31 (11.7)	489 (11.5)	
No	3999 (88.5)	234 (88.3)	3765 (88.5)	
History of cancer treatment n (%)				0.41
Yes	362 (8.0)	17 (6.4)	345 (8.1)	
No	4157 (92.0)	248 (93.6)	3909 (91.9)	

* Statistical significance with *p*-value ≤ 0.05 shown in bold.

**Table 2:** Multivariable Cox regression model. Summary for immediate and delayed implant placement for the characteristics evaluated.

Variable	Hazzard Ratio	95% Confidence Interval		*p*-Value *
		Lower	Upper	
Treatment: immediate implant	1.61	0.57	4.57	0.37
Age (increase by 1 year)	1.00	0.97	1.02	0.70
Gender: male	2.06	1.08	3.92	**0.03**
Tobacco use	1.76	0.88	3.54	0.11
Socioeconomic status: high	0.62	0.32	1.20	0.15
Insurance status: insured	1.19	0.68	2.08	0.55
Arch: mandible	1.02	0.59	1.78	0.94
Region: posterior	1.40	0.72	2.71	0.32
Hypertension	0.99	0.49	1.97	0.97
History of heart attack	0.00	0.00	-	0.98
Hypercholesterolemia	0.89	0.44	1.81	0.75
Asthma	0.00	0.00	5.205 × 10^293^	0.97
Diabetes	0.38	0.09	1.64	0.19
Thyroid disorder	0.60	0.21	1.72	0.34
Kidney disease	2.24	0.49	10.22	0.30
Arthritis	1.35	0.67	2.72	0.40
Artificial joint	1.36	0.50	3.71	0.55
Osteoporosis	4.56	1.80	11.54	**0.001**
Depression	1.70	0.74	3.92	0.21
Anxiety	1.22	0.47	3.16	0.69
Cancer	2.01	0.60	6.71	0.26
History of cancer treatment	0.31	0.06	1.56	0.15

* Statistical significance with *p*-value ≤ 0.05 shown in bold.

## Data Availability

Data available on request from the authors.
